# Dynamics and Mechanism of Off‐ to On‐Switching in Dreiklang a Decoupled Reversibly Switchable Fluorescent Protein

**DOI:** 10.1002/anie.202518264

**Published:** 2025-11-10

**Authors:** Anam Fatima, Yongle He, Danielle Rosenberger, Gregory M. Greetham, Partha Malakar, Andras Lukacs, Peter J. Tonge, Stephen R. Meech

**Affiliations:** ^1^ School of Chemistry University of East Anglia Norwich NR4 7TJ U.K; ^2^ Department of Chemistry Stony Brook University Stony Brook New York 11794 USA; ^3^ Central Laser Facility, Research Complex at Harwell Rutherford Appleton Laboratory Didcot OX11 0QX U.K; ^4^ Department of Biophysics, Medical School University of Pecs Pecs 7624 Hungary

**Keywords:** Biophysics, Fluorescent protein, Infra‐red, Photophysics, Photoswitching, Ultrafast

## Abstract

Dreiklang is a reversibly switchable (rs) fluorescent protein (FP) with a unique off‐state, a UV absorbing hydrated form of the typical FP chromophore. Here we report ultrafast dynamics of the off‐ to on‐state transition in Dreiklang using complementary ultrafast optical and vibrational transient absorption to resolve chromophore driven protein structural dynamics. This approach allows observation of the real‐time response in a protein to bond breaking and forming events. The excited electronic state decays in a nonsingle exponential fashion in tens to hundreds of picoseconds, undergoing photodehydration with a yield of several per‐cent. The primary photoproduct formed is identified as the cis protonated form of the FP chromophore, initially in a perturbed H‐bonded environment. This primary product relaxes on a few microseconds timescale by a mechanism involving changes to a glutamic acid residue and modifications of the amide backbone, possibly involving a carbonyl to imine tautomerization. The temporal and spectral resolution of Dreiklang's photodehydration provides data against which to test quantum chemical calculations of reaction dynamics in proteins and suggests a route to modifying and potentially enhancing its photoswitching properties.

## Graphical abstract

Dreiklang is a reversibly switchable fluorescent protein with a unique photohydration/dehydration mechanism. Using complementary transient absorption and femtosecond—microsecond transient IR, we follow the off‐to‐on photo‐dehydration. A multistep process is revealed, where a hundreds‐of‐picoseconds photoreaction is followed by microsecond structural reorganization, providing new mechanistic insight.

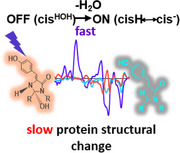

## Introduction

**Figure 1:**
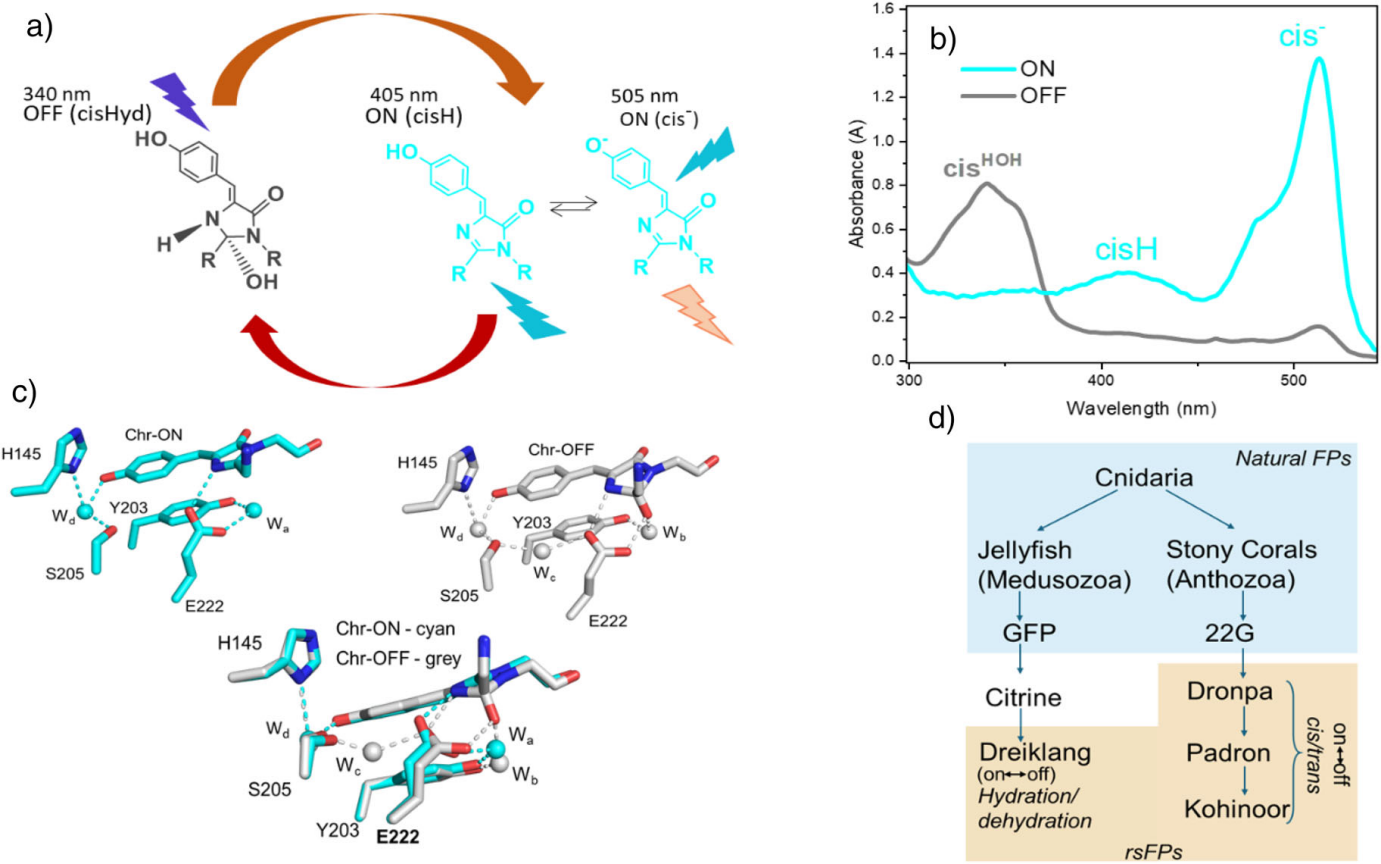
a) Mechanism and molecular structures of the off‐ and on‐states of Dreiklang. b) Electronic absorption spectra of Dreiklang in Tris‐HCl buffer at pH 6.5 before irradiation where off‐state (340 nm cis^HOH^ band) and the on‐state equilibrium (400 nm cisH and 488 nm cis^−^) coexist and after irradiation at 405 nm, where the off‐state dominates. c) chromophore structure and environment for the on (PDB 3ST4; cyan) and off (PDB: 3ST3; grey) states of Dreiklang plus an overlay of the dreiklang structures in the ON and OFF states. d) Evolutionary origins and distinct switching mechanism of Dreiklang compared to other rsFPs; hydration/dehydration versus cis/trans isomerization and proton transfer.

Reversibly switchable fluorescent proteins (rsFPs) can be photochemically converted between bright (fluorescent) and dark states by irradiation at different wavelengths.^[^ ^1^, ^2^ ^]^ This property has made them a critical tool in the development of various modes of super‐resolution bioimaging and has applications in other fields such as optogenetics.^[^ ^3^ ^]^ The rsFP Dreiklang,^[^ ^4^ ^]^ obtained by mutation of Citrine (a yellow fluorescent protein), occupies a unique position among rsFPs because its off‐state is a photochemically generated chemically distinct hydrated form of the chromophore in its cis isomer (cis^HOH^, Figure 1a) as opposed to the trans isomer found in both negative and positive switching rsFPs exemplified by Dronpa and Padron respectively.^[^ ^5^, ^6^ ^]^ One consequence of hydration is disruption of the extended conjugation in the chromophore, which results in an off‐state that absorbs in the UV (340 nm, Figure 1b). Irradiation at 340 nm then leads to photodehydration and reformation of the protonated FP chromophore (cisH), yielding the on‐state, which ultimately forms an equilibrium with the deprotonated (anionic) form cis^−^ (Figure 1a). Subsequent irradiation of cis^−^ near 500 nm yields steady fluorescence while irradiation of cisH near 400 nm gives rise to the on‐ to off‐state photohydration reaction; the reversible on/off conversion can be repeated over many cycles.^[^ ^4^ ^]^ Thus, the three transitions in Dreiklang (emission and on/off photoconversion) are driven by three distinct wavelengths, so it has been labeled a decoupled rsFP.^[^ ^1^ ^]^ Dreiklang has found applications in REversible Saturable OpticaL Fluorescence Transitions (RESOLFT) nanoscopy,^[^ ^7^ ^]^ and its multiwavelength switching has potential in multicolour frequency modulation imaging.^[^ ^7^, ^8^ ^]^

The protein structure of the off and on states of Dreiklang were determined by correlating electronic spectra with crystal structures measured after irradiation with blue/UV light to make the off/on states respectively (Figure 1c);^[^ ^4^ ^]^ the corresponding solution spectra are shown in Figure 1b. The structures revealed that generation of the hydrated form is accompanied by relocation of the adjacent E222 residue and that the water molecules had been swapped (the one used during hydration, W_a_ being replaced in the final off‐state structure by W_b_). The quantum yield for the on‐ to off‐ transition was measured later as 0.4%.^[^ ^9^ ^]^ The off to on yield has not been reported, but Brakemann et al. noted that complete off‐ to on‐conversion could be achieved with ca. 100 fold lower irradiance than required for on‐ to off‐switching, suggesting a yield exceeding 10%. The mechanism of on‐ to off‐state switching (excitation at 400 nm) was investigated in detail by ultrafast transient absorption (TA) spectroscopy coupled with mutagenesis and quantum chemical calculations.^[^ ^9^, ^10^, ^11^, ^12^ ^]^ In that case, the primary process in the photohydration reaction (Figure 1a) was identified as tyrosine to excited chromophore electron transfer, although other processes occur in parallel. In contrast, the UV driven off‐ to on‐state transition has received much less attention, although there is a quantum chemical calculation of the thermal (ground state) reaction pathway.^[^ ^13^ ^]^ The photohydration of Dreiklang is a particularly interesting case for ultrafast spectroscopy, not only for the intrinsic interest in Dreiklang switching but also because it affords a rare opportunity to observe in real‐time the consequences of a covalent bond breaking and making reaction in a protein. In this work, we present an experimental study of the off‐ to on‐state photochemical dehydration reaction, utilizing complementary ultrafast TA and femtosecond to millisecond time resolved infra‐red (TRIR) spectroscopy. Both methods resolve the picosecond excited state dynamics of cis^HOH^, while TRIR also resolves ground state dynamics and the structural evolution in the protein, which occur as the on‐state begins to form on the nanosecond to microsecond timescale. The present results suggest that the initial photodehydration of cis^HOH^ proceeds in the excited state to rapidly (tens to hundreds of picoseconds) generate the cisH form of the chromophore, followed by ground state structural changes in a few microseconds involving the glutamic acid residue (E222) in an H‐bonded network adjacent to the chromophore accompanied by slower changes in protein backbone and chromophore structure.

## Results and Discussion

### Transient Absorption

**Figure 2:**
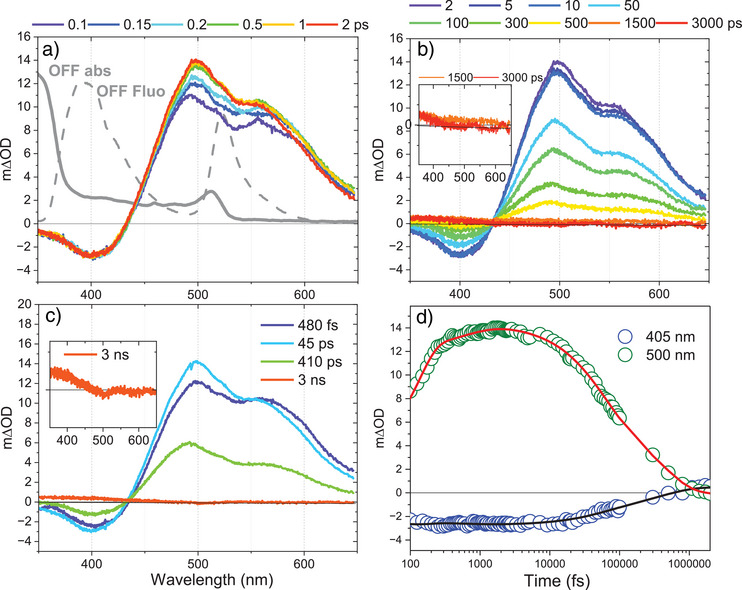
Transient absorption following 340 nm excitation of cis^HOH^ in D_2_O tris buffer pD 6.5. a) Evolution over the first 2 ps. The steady state absorption (grey solid) and emission (grey dashed) following 340 nm excitation are shown to aid assignment of the negative signal to stimulated emission (weak emission from 340 nm excitation of a small amount of the highly emissive on‐state cis^−^ at 521 nm is also detected). b) Evolution of TA from 10 ps to 3 ns. c) Evolution associated difference spectra (EADS) obtained from the global analysis of the TA data. The insets in (b) and (c) show product formation as absorption at ca 400 nm. d) The quality of the global fit at two critical wavelengths is shown (see text).

Figure 2 shows evolution of the TA of Dreiklang in D_2_O buffer following off‐state excitation with 100 fs pulses at 340 nm. Measurements were made in a flow cell in which the reservoir was continuously irradiated at 405 nm to retain a dominant off‐state population. Full experimental details are provided in the Supporting Information. The TA is a pump‐on minus pump‐off difference spectrum and has two dominant features, a strong bimodal transient absorption (positive) signal peaking at 500 nm and a weaker negative signal at 405 nm assigned to stimulated emission (SE), as confirmed by comparison with the steady state emission spectrum excited at 340 nm (Figure 2a). After an initial subpicosecond increase in amplitude and associated reshaping of the transient feature (see also Figure 2d), the dominant kinetics are a nonsingle exponential decay of TA and SE in a concerted fashion. This is consistent with the decay of excited cis^HOH^ to largely repopulate the ground state on the tens to hundreds of picosecond time scale. In addition, a long lived (>> 3 ns) ground state product appears, with an absorption maximum near 400 nm (Figure 2b inset).

All these features are captured in the global analysis. A three‐step scheme with a final time independent spectrum was required to fit the data. The evolution associated difference spectra (EADS) arising from this sequential mechanism are shown in Figure 2c with those from the corresponding parallel decay (D) mechanism (the DADS) shown in the Supporting Information (Figure S1). The sub‐picosecond increase in the TA amplitude is inconsistent with population kinetics so is assigned to a rapid change in electronic structure after excitation of the Franck–Condon state of cis^HOH^, to yield a relaxed excited state with an increased S_1_ → S_n_ transition moment, cis^HOH^*. This evolution is accompanied by a reshaping of the TA. The subsequent relaxation kinetics of cis^HOH^* are modelled by a 45 ps relaxation phase in both TA and SE plus a slower (410 ps) component of lower amplitude, suggesting inhomogeneity in the cis^HOH*^ decay kinetics. These tens to hundreds of ps lifetimes represents exceptionally long excited state lifetime for an rsFP off‐state, compared to Dronpa, Padron, and their mutants. Both of those rsFPs undergo a cis‐trans isomerization, which results in quenching of the excited electronic state in a few to tens of picoseconds.^[^ ^14^, ^15^, ^16^, ^17^ ^]^ This illustrates the unique properties of Dreiklang cis^HOH^, which may reflect its distinct evolutionary pathway (Figure 1d, although all the common rsFPs are themselves mutations of wild type FPs or chromoproteins). Measurements here are reported in D_2_O buffer. Identical TA measurements in H_2_O buffer yield the same lifetimes, so there is no evidence for a deuterium isotope effect on the kinetics (Figure S2). The cis^HOH^* decay ultimately yields a product absorption, which peaks at 400 nm and does not decay on the 3 ns timescale of our TA measurement. This is consistent with photochemical formation of either cisH or possibly the cationic form of the FP chromophore cisH_2_^+^ by nonsingle exponential photodehydration of cis^HOH^*; cisH_2_^+^ is mentioned as it plays a role in the proposed ground state dehydration reaction.^[^ ^13^ ^]^ Both these forms of the chromophore absorb near 400 nm.^[^ ^18^, ^19^ ^]^ The readily observable product state absorption is consistent with an estimated yield for off‐ to on‐state photoconversion of several %. Significantly, there is no corresponding cis^−^ absorption (expected at >480 nm) even though it has the larger oscillator strength (Figure 1b). Thus, the deprotonation to establish the on‐state equilibrium (Figure 1a) occurs on a time scale longer than 3 ns.

### Transient Vibrational Spectroscopy

**Figure 3:**
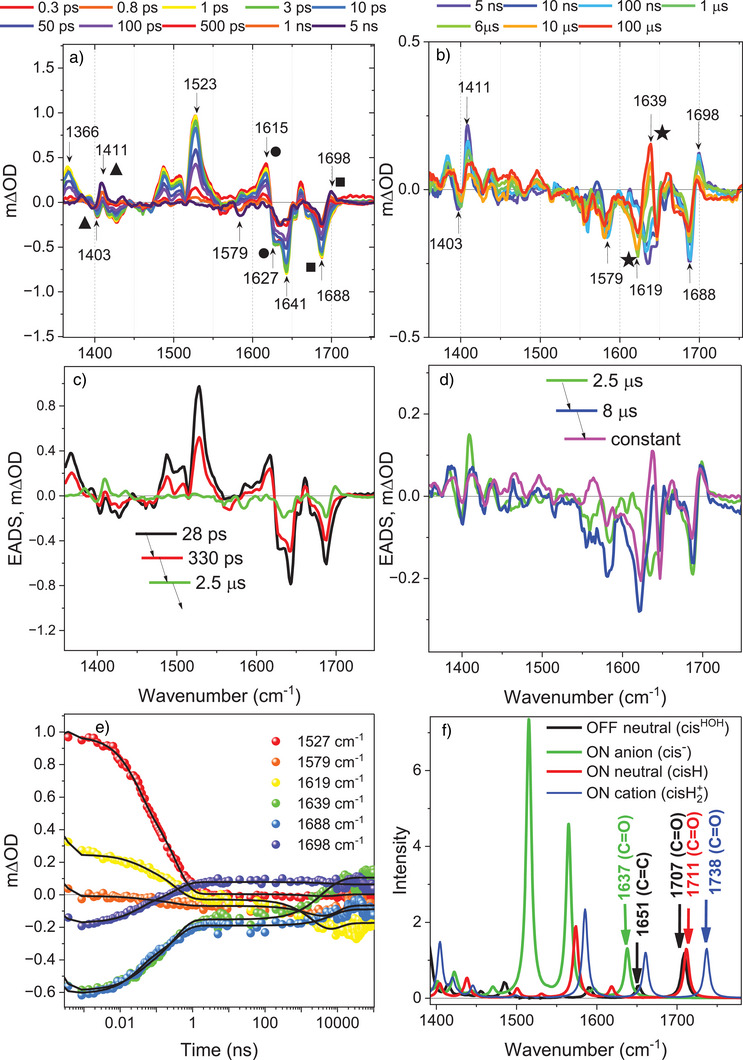
TRIR of Dreiklang after off‐state excitation at 340 nm in D_2_O buffer, pD 6.5. a) TRIR data recorded from 0–5 ns. b) TRIR recorded from 5 ns to 100 µs. Global analysis of the complete TRIR data required five sequential components at 28 ps, 330 ps, 2.5 us, and 8 us plus a long‐lived component as shown in c) and d). (c) Earliest three of five EADS recovered from global analysis over the entire time range, to characterize the cis^HOH^* decay, with the associated time constants. (d) as (c) showing the final three of five EADS characterizing the slower dynamics. e) Fit from global analysis (five sequential components plus a long‐lived component) of kinetic data presented at selected wavelengths. f) DFT calculated infra‐red transition wavenumbers for various charged states and hydration forms of the chromophore ground state. All calculated wavenumbers are scaled by the recommended correction factor 0.957 for comparison with experimental data.

The TRIR data are shown on sub‐ps to ns (Figure 3a) and ns to one hundred µs (Figure 3b) time scales. Prominent bleaches (negative signals) at 1688 and 1641 cm^−1^ appear promptly on excitation (within 200 fs). These are well reproduced in DFT calculations of the ground state IR spectrum of the cis^HOH^ and can thus be assigned to C═O and C═C methine bridge stretches (calculated at 1707 cm^−1^ and 1651 cm^−1^, respectively) (Figure 3f). It is interesting to note that the hydrated spectrum is quite different to that of cisH form of the chromophore, with a much less prominent contribution from the C═C and phenyl ring modes. Presumably these changes reflect the new electronic structure. There is also a bleach in the measurement at 1627 cm^−1^ (●), which has no counterpart in the DFT calculation but does have a corresponding transient at 1615 cm^−1^ (●). We assign this to a mode of the protein that is strongly coupled to the electronic excitation and promptly downshifted in wavenumber upon excitation of cis^HOH^; similar effects are seen in other rsFPs.^[^ ^20^ ^]^ There are also a number of promptly formed transients notably at 1523 and 1366 cm^−1^, which we ascribe to cis^HOH^* rather than to protein modes, as they have no nearby corresponding ground state. Confirmation of this assignment requires calculations of excited state frequencies of cis^HOH^*. Again, we note that the excited state TRIR spectrum of Dreiklang is very different to that of the cisH FP chromophore, which exhibits only weak excited state vibrational transitions.^[^ ^21^, ^22^ ^]^

The decay of cis^HOH^* (1523 cm^−1^) and refilling of the ground state (e.g., 1688 cm^−1^) on the tens to hundreds of picosecond time scale, as also seen in TA (Figure 2b), is accompanied by an evolution in the TRIR spectral profile, most notably in the bleach near 1641 cm^−1^ assigned to a protein–chromophore coupled mode. After 1 ns a vibrational difference spectrum associated with the initial product formation is observed, with notable contributions from a bleach/transient pair yielding a “differential” lineshape at 1688/1698 cm^−1^(■), a similar pair at 1403/1411 cm^−1^ (▲) and a bleach at 1579 cm^−1^. However, the evolution of these primary product modes continues on a ns to 100 µs time scale (Figure 3b), long after cis^HOH^* has decayed. Even though Figure 3b focuses on small amplitude changes in the tens of μOD range, the evolution in the 1688/1698 cm^−1^ and 1403/1411 cm^−1^ pairs over tens of microseconds are obvious and well resolved. Both pairs show complex evolution, with negative and positive lobes exhibiting different kinetics, suggesting multiple underlying contributions. The 1688/1698 cm^−1^ pair persists beyond 100 µs. As the microsecond time scale relaxation proceeds, a new and prominent bleach/transient pair appears at 1619/1639 cm^−1^ (★) developing over several microseconds, which we assign to changes in protein structure around the chromophore, specifically a spectral blue‐shift in an amide I mode.

The global analysis data assuming a sequential model are shown in Figures 3c,d. To adequately fit the full‐time range (for fit quality see Figure 3e) five first order steps and a final component were required. The EADS are shown in Figures 3c,d and the corresponding DADS are in the Figure S3. As described below, the EADS seem the more appropriate description here. The first two EADS largely represent the excited state decay (e.g., transients at 1523 and 1366 cm^−1^, both of which decay to the baseline) in tens and hundreds of ps, consistent with TA data (Figure 2b,c). Data earlier than 200 fs are compromised by pulse overlap artefacts, so the sub picosecond component seen in TA is not clearly resolved. However, if the sub‐picosecond kinetics from the TA are imposed on the TRIR global analysis, no new spectral features are recovered (Figure S4), suggesting the sub picosecond evolution in electronic structure does not modify significantly the vibrational spectrum of the cis^HOH^*. The picosecond EADS have time‐constants (28 ps and 330 ps) for the excited state relaxation, matching those observed in the TA (45 ps and 410 ps, Figure 2c). The two subnanosecond EADS are similar and dominated by modes assigned to cis^HOH^/cis^HOH^*, but there is a subtle evolution in the complex band near 1640 cm^−1^, which was assigned above to a coupled protein–chromophore mode. This suggests a tens of ps evolution in the protein mode coupled to the electronic transition occurring on a timescale faster than the hundreds of ps excited state population relaxation; a similar observation was reported in the positive rsFP Kohinoor.^[^ ^23^ ^]^ An equivalent parallel model for cis^HOH^* decay would be an inhomogeneous arrangement of chromophore–protein interactions decaying with different decay times.

As the excited state decays, signals near 1688/1698 and 1403/1411 cm^−1^ develop as pairs along with a 1579 cm^−1^ bleach. The subsequent complex kinetics of these product modes are represented by two EADS on the microsecond time scale and one permanent contribution. The appearance of these signals long after excited state decay supports the sequential model. We note that global analysis imposes a multistep mechanism on what may be more complex dispersive kinetics, often observed in protein relaxation. Here, we have fit to the minimum number of components required to obtain a good statistical description of the data (Figure 3e) and ensured that addition of further component does not uncover any new spectral features. The times associated with each EADS thus reflect time constants characterizing that spectral evolution rather than state to state kinetics. We cannot rule out a more complex underlying dispersive kinetics,^[^ ^24^, ^25^, ^26^, ^27^, ^28^ ^]^ and experimentally the two can be difficult to distinguish.^[^ ^29^ ^]^

The first ground state relaxation occurs in 2.5 µs and sees decreasing amplitude in the cis^HOH^ bleach mode at 1641 cm^−1^ and initial formation of the 1619/1639 cm^−1^ pair associated with perturbation of an amide I mode. There is also a deepening bleach near 1579 cm^−1^ and a decrease in amplitude at 1411 cm^−1^. On a longer time scale, the 8 µs EADS mainly shows further evolution in the amide I mode pair along with a partial decay of the 1698 cm^−1^ transient and a filling of the 1579 cm^−1^ bleach. The kinetics associated with these specific modes are shown in Figure 3e. All this leaves a final EADS, here assigned a constant lifetime, although comparison with a light minus dark steady state IR difference spectrum will reveal if further structure changes occur on the millisecond timescale. We note for example that the final EADS does not show a prominent product absorption near 1500 cm^−1^, which is expected for cis^−^ formation, the dominant form in the on‐state equilibrium (Figure 1b). This observation suggests that deprotonation and on‐state equilibration occur on a longer timescale than our hundreds of µs time window.

### Mechanism

The primary process in the excited state decay of cis^HOH^* is branching to the initial ground state and to a metastable product. The product is assigned as photochemical bond breaking to leave a new form of the chromophore (cisH or cisH_2_^+^) and a perturbed protein environment. This is consistent with the ground state calculation of Grigorenko et al., who calculated that the dehydration reaction has a high activation barrier in the ground state, so electronic excitation is required for efficient photoconversion.^[^ ^13^ ^]^ There are a number of possible contributions to the 1688/1698 cm^−1^ product pair formed from cis^HOH^* decay (Figure 3a,c). At least part of the 1688 cm^−1^ bleach must arise from the fraction of the ground state not repopulated due to product formation. In that case, a possible contribution to the 1698 cm^−1^ transient is a corresponding blue shifted carbonyl stretching mode of the product state of the chromophore formed on photodehydration (as also seen in the ca 400 nm TA product absorption). In addition, protonation of the E222 residue adjacent to the reaction site and H‐bonded to water molecules involved in the hydration/dehydration reaction (Figure 1c) could also contribute to the 1698 cm^−1^ transient. Indeed, the 1688/1698 cm^−1^ lineshape evolution shows differential evolution of bleach and transient parts (Figure 3d inset) suggesting multiple underlying contributions.

The DFT calculations of the IR absorption of the different charge and hydration forms of the chromophore (Figure 3f) suggest a 4 cm^−1^ upshift in carbonyl stretch upon formation of cisH from cis^HOH^, whereas there is a 31 cm^−1^ upshift for the carbonyl upon cisH_2_^+^ formation. In contrast, the cis^−^ carbonyl is downshifted by 70 cm^−1^ so is not a candidate for the 1698 cm^−1^ signal (which is consistent with the absence of cis^−^ in the TA product, Figure 2c). The cisH_2_^+^ state plays a key role as a primary intermediate in the calculated thermal mechanism of Grigorenko et al.^[^ ^13^ ^]^ However, the calculated upshift on carbonyl wavenumber is significantly larger than observed, suggesting that this intermediate, if formed, must be short lived. A short lifetime is not unexpected, given that cisH_2_^+^ has a p*K*_a_ of 1.4^[^ ^19^ ^]^ suggesting it would be unstable with respect to deprotonation to form cisH. In contrast, the 1698 cm^−1^ transient is stable for tens of microseconds. Thus, the calculated 4 cm^−1^ upshift between cis^HOH^ and cisH fits the observations better, and that state must then be formed as the cis^HOH^* decays.

The photodehydration reaction, which forms a water molecule and cisH (and must also displace another water from an adjacent site of lower occupancy^[^ ^4^ ^]^) will necessarily lead to a rearrangement of the H‐bonding network between the chromophore, E222, and the Y203 (Figure 1c). Protonation of the carboxylate side chain of E222 would also lead to formation of a carbonyl transient above 1700 cm^−1^, as was observed in the GFP excited state proton transfer reaction.^[^ ^30^ ^]^ Thus, E222 protonation could also give rise to the 1698 cm^−1^ transient. However, Glu protonation should be accompanied by bleaches near 1400 and 1600 cm^−1^ associated with loss of carboxylate modes,^[^ ^31^ ^]^ which was not observed either in the third EADS or later. Another possibility is that E222 is already protonated in the ground state (the p*K*_a_ is 4.5 in water but the anion may be less stable in the protein environment). In that case, the decrease in amplitude of the 1688 cm^−1^ product state bleach observed on the slower microsecond time scale may indicate changes in the H‐bonding environment of the E222 carbonyl, modifying its extinction coefficient. However, on the several microseconds timescale, the main spectral change is the development and then shift in the amide I band (1619/1639 cm^−1^). This signal appears in 2.5 µs and then evolves further in 8 µs. Also, in the 8 µs step, there is an additional filling of the 1579 cm^−1^ bleach and formation of a new transient at 1565 cm^−1^ in the final spectrum. These changes suggest that the photodehydration reaction and subsequent relaxation in the immediate environment of the chromophore propagates away from the hydration site, leading to wider changes in protein backbone structure, as well as to E222. Large changes in backbone structure are not apparent in the on/off crystal structure (Figure 1c). A more subtle effect on the amide I spectrum could be an imine/carbonyl shift, which would lead to the observed up‐shift and such a change features in the mechanism of Grigorenko et al.^[^ ^13^ ^]^ This change occurs on the several microsecond timescale and will be accompanied by changes in the H‐bond network.

## Conclusion

Dreiklang has a unique photoswitching mechanism among rsFPs. While both the negative (e.g., Dronpa) and positive (e.g., Padron) rsFPs rely upon cis/trans photoisomerization as the primary step in their off/on switching mechanisms, Dreiklang undergoes a photo hydration/dehydration reaction. A consequence of this unique mechanism is the formation of a new hydrated chromophore off‐state absorbing in the UV (340 nm). Consequently, in Dreiklang the on‐, off‐, and fluorescence excitation steps are all initiated by distinct irradiation wavelengths, unlike in Dronpa and Padron where on‐ and off‐ or on‐ and fluorescence excitation respectively occur at a common wavelength. This “decoupling” of all the key photoprocesses gives the experimenter independent control, which has proven useful in RESOLFT superresolution measurements and is attractive for amplitude or frequency modulation based imaging experiments. Here we have probed the photochemical mechanism of Dreiklang with ultrafast optical experiments and fs to 100 µs transient IR. The transient IR allows us to probe mechanism in the off‐ to ‐on‐switching in Dreiklang at times after the chromophore excitation has died away but while the spectroscopy of the protein evolves as the structure reaches towards the on‐state. As well as affording us the rare opportunity of studying a chemical reaction in a protein in real‐time, these measurements provide mechanistic details which will be useful in designing mutations to control the rate and yield of photoswitching.

**Figure 4:**
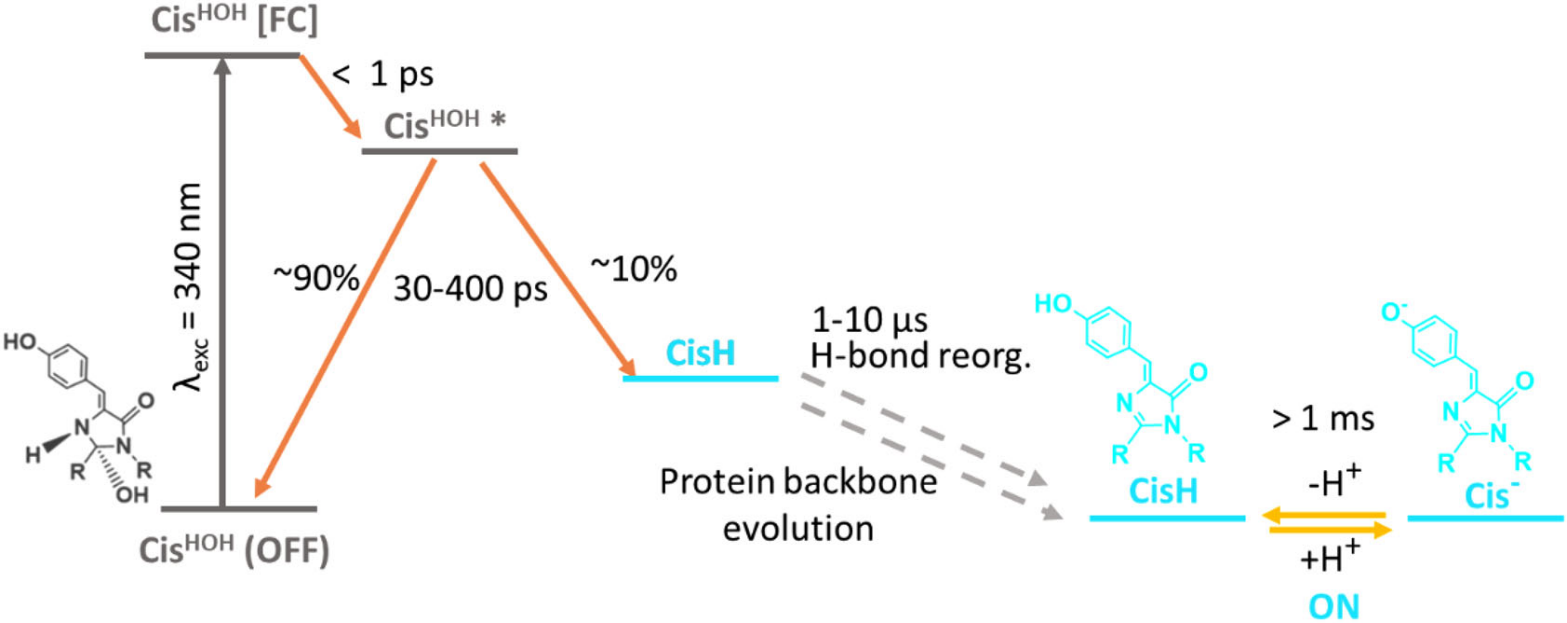
Proposed mechanism for Dreiklang off‐ to on‐state switching with associated yields and observed time constants.

The mechanism for the off‐to on‐state transition of Dreiklang which emerges from the time resolved spectroscopy is illustrated in Figure 4. The primary step following electronic excitation and a sub ps evolution in electronic structure (Figure 2a) is that the resulting cis^HOH^* chromophore relaxes on a sub nanosecond timescale to form a cisH ground state in an unrelaxed H‐bond environment involving at least the chromophore and E222 residue. This new H‐bonding environment relaxes initiating a multi‐timescale structural relaxation in adjacent amide I modes, which occurs over several microseconds to yield a long‐lived product state. This “final” state must then relax to the on‐state equilibrium in >0.1 ms (the latest time point observed) by which time the 1500 cm^−1^ mode characteristic of the on‐state anion had not been resolved.

It is interesting to compare these observations with the ground state calculations of Grigorenko et al.^[^ ^13^ ^]^ In their mechanism, the initial intermediate formed after dehydration is the cisH_2_^+^ chromophore, which relaxes over a low barrier to yield cisH and protonated E222. In our study we do not detect cisH_2_^+^, but this may be for kinetic reasons, due to the high reactivity of cisH_2_^+^ in its environment (see above), which is consistent with the low barrier calculated. Further, it is likely that the photoreaction delivers the system to a different point on the reactive potential surface compared to the thermal reaction, bypassing the reactive cisH_2_^+^ intermediate. A more significant difference between calculation and experiment is the requirement in the calculations for protonation of E222. In the TRIR data, there is no evidence for the expected bleaches associated with the disappearance of symmetric/asymmetric pair of carboxylate stretches. Thus, we propose a mechanism mediated by a protonated E222 involved in a complex H‐bonded environment with the chromophore and adjacent water molecules. This protonated E222 is perturbed by the changes in H‐bond structure upon cisH formation, contributing to a complex line shape evolution in the carbonyl stretch region. Significantly, the calculations find an important role for the amide backbone in the later stages of the reaction. This feature is in good agreement with the present data, which point to a perturbation of amide I modes on the microsecond timescale as the new H‐bond environment relaxes. Indeed, the shift observed experimentally is to higher wavenumber, which matches with the carbonyl to imine shift which plays a role in the calculations.

Further testing of this mechanism will be achieved by studies of Dreiklang mutants in the key E222, S205, and H145 residues (Figure 1c). The E222Q mutation does not show photohydration. In that case, a more detailed assignment of transient IR spectra will be possible through study of ^13^C isotope edited proteins.^[^ ^21^ ^]^ Such measurements are planned, and together with additional quantum mechanical and molecular mechanics calculations will provide detailed insights into the real‐time dynamics of this intriguing intraprotein reaction.

## Conflict of Interests

The authors declare no conflict of interest.

## Supporting information



Supporting Information

## Data Availability

The data that support the findings of this study are available from the corresponding author upon reasonable request.
