# Supplementary material for: Dynamics and Mechanism of Off‐ to On‐Switching in Dreiklang a Decoupled Reversibly Switchable Fluorescent Protein
Source: Angew Chem Int Ed Engl. 2025 Nov 10;65(1):e18264. doi: 10.1002/anie.202518264 (PMC12707319; doi:10.1002/anie.202518264)
Supplement: Supplementary file 1 — Supporting Information [file ANIE-65-e18264-s001.docx]

**SUPPLEMENTARY INFORMATION**

**Dynamics and Mechanism of Off- to On-switching in Dreiklang a Decoupled Reversibly Switchable Fluorescent Protein**

Anam Fatima,^1^ Yongle He,^2^ Danielle Rosenberger,^2^ Gregory M. Greetham,^3^ Partha Malakar,^3^ Andras Lukacs,^4^* Peter J. Tonge^2^* and Stephen R. Meech^1^*

*^1^School of Chemistry, University of East Anglia, Norwich NR4 7TJ, U.K; ^2^Department of Chemistry, Stony Brook University, Stony Brook, New York 11794, United States; ^3^Central Laser Facility, Research Complex at Harwell, Rutherford Appleton Laboratory, Didcot OX11 0QX, U.K. ^4^Department of Biophysics, Medical School, University of Pecs, 7624 Pecs, Hungary.*

Contents

[**1.** **Experimental section** 1](#_Toc204973842)

[**a.)** **Steady-State Absorption and Emission** 1](#_Toc204973843)

[**b.)** **Visible transient absorption (TA)** 1](#_Toc204973844)

[**c.)** **Time-resolved Infra-red spectroscopy (TRIR)** 2](#_Toc204973845)

[**d.)** **Time-resolved Multiprobe spectroscopy (TRMPS)** 2](#_Toc204973846)

[**e.)** **Cloning, protein expression and purification** 3](#_Toc204973847)

[**2.** **Figures** 4](#_Toc204973848)

[Figure S1: Decay-associated difference spectra (DADS) obtained from global analysis of the OFF-state (cisHOH) transient absorption data using a parallel decay model. 4](#_Toc204973849)

[Figure S2: Kinetic traces at 500 nm from transient absorption measurements in H₂O (pH 6.5) and D₂O (pD 6.5), shown overlaid for comparison. 5](#_Toc204973850)

[Figure S3: The EADS recovered for TRIR when the sub ps component from TA is imposed. No new spectral features are revealed. 5](#_Toc204973851)

[Figure S4: Decay-associated difference spectra (DADS) obtained from global analysis of the OFF-state (cis^HOH^) transient absorption data using a parallel decay model. 6](#_Toc204973852)

[**References** 7](#_Toc204973853)

# **Experimental section**

## **Steady-State Absorption and Emission**

**UV–Visible absorption spectra** in the ground state were recorded using a **PerkinElmer Lambda XLS spectrophotometer**, employing **quartz cuvettes with a 1 cm optical path length.** The sample concentration was maintained below **1 μM** to ensure an absorbance below 0.1. **Steady-state fluorescence emission spectra** were measured at room temperature on an **Edinburgh Instruments FS5 spectrofluorometer** using a **right-angle detection geometry** and a **2 nm bandwidth** for both excitation and emission.

## **Visible transient absorption (TA)**

The transient absorption setup employed in this study has been described in detail elsewhere^[1]^. Briefly, both pump and probe beams were derived from the 800 nm fundamental output of a Spectra-Physics Mai Tai laser oscillator, which was amplified using a Ti:Sapphire regenerative amplifier (Spectra-Physics Spitfire ACE). The resulting pulses had a central wavelength of 800 nm, a pulse duration of 100 fs, a repetition rate of 1 kHz, and an energy of 5 mJ per pulse. These pulses were used to drive an optical parametric amplifier (OPA, Light Conversion TOPAS Prime), producing a tunable pump beam for sample excitation.

A white-light continuum (WLC) probe was generated by focusing a portion of the 800 nm fundamental beam onto a 3 mm CaF_2_ window (which was translated bi-axially to prevent damage), yielding a broadband spectrum spanning 350–750 nm. Transient absorption spectra were collected following excitation at 340 nm. The 340 nm pump beam energy at the sample position was attenuated to 100 μW (equivalent to 100 nJ per pulse).

All experiments were carried out in 1 mm pathlength flow cuvettes, with optical densities kept below 1 (corresponding to concentrations under 200 μM). To maintain the sample in its OFF state, the sample reservoir was continuously illuminated with a 405 nm LED.

## **Time-resolved Infra-red spectroscopy (TRIR)**

Time-resolved infrared (TRIR) spectroscopy was conducted using LifeTime systems at the Central Laser Facility within the Research Complex at Harwell^[2]^. Transmitted light from the sample was measured with pump-on and pump-off, employing a visible pulse to excite the sample, followed by an infrared probe to capture the vibrational spectrum. The sample was excited with 340 nm light pulses (200 nJ) at a repetition rate of 1 kHz, yielding high signal-to-noise spectra with approximately 150 fs resolution. Protein sample, prepared at a concentration of 1–2 mM in D_2_O, were analyzed using a 50 µm path length CaF_2_ cell. To reduce photobleaching and sample degradation, the sample cell was rastered while the samples were flowed through the cell at a rate of approximately 1.5 mL/min. The spectra were calibrated against the IR spectrum of polystyrene. To maintain the sample in its original OFF-state, the reservoir was continuously illuminated with a 405 nm LED.

## **Time-resolved Multiprobe spectroscopy (TRMPS)**

TRMPS spectra were obtained from 100 fs to 200 µs at the STFC Central Laser Facility. The TRMPS method has been described previously^[3]^, and used by us to study the photoswitching in several rsFPs. This method enables time resolved IR spectra to be acquired over 10 decades of time from sub-ps to ms. The sample was analysed using a flow cell, and data were acquired using a 340 nm pump operated at 0.6-0.8 µJ per pulse and a repetition rate of 1 kHz. After the measurements were recorded, the extent of photoconversion was shown to be negligible using absorbance spectroscopy. The spectral resolution was 3 cm^-1^ and the temporal resolution was 200 fs. A typical measurement was acquired during 45 min of data collection. All samples were prepared at 1-2 mM concentration in D_2_O buffer. Spectra were calibrated relative to the IR transmission of a pure polystyrene standard sample placed at the sample position.

## **Cloning, protein expression and purification**

The codon-optimized gene encoded Dreiklang (1-239) was synthesized and cloned into the pET15b vector in frame with an N-terminal six-His tag using the NdeI and BamHI restriction sites (Genscript). The final construct was confirmed by Sanger Sequencing. Protein expression was conducted by transforming the pET15 Dreiklang plasmid into *E.coli* BL21 (DE3) cells. A single colony was used to inoculate 10 mL 2x-YT media containing 100 μg/mL of ampicillin and shaken overnight at 37 °C. The 10 mL overnight culture was used to inoculate 1 L of 2x-YT media containing antibiotic in a 4 L flask, which was then shaken at 37 °C (250 RPM) until the optical density (OD_600_) reached ~0.8. The temperature was lowered to 18 °C, and 1 mM isopropyl β-D-1-thiogalactopyranoside (IPTG, Gold Biosciences) was added to induce protein expression. The cells were harvested after 16 h by centrifugation at 5,000 RPM (6,238 x g; 4 °C) for 20 min, and the cell pellet was stored at −20 °C until needed.

The cell pellet containing each protein was thawed and resuspended in lysis buffer (20 mM Tris pH 8, 150 mM NaCl, and 5 mM imidazole) and lysed by sonication. The cell debris was removed by ultracentrifugation at 40,000 RPM (185,511 x g) for 1 h at 4 °C. After the ultracentrifugation, the supernatant was loaded onto a 3 mL Ni-NTA column, which was then washed with 10-20 column volumes of lysis buffer containing 20 mM imidazole and the protein was eluted with 500 mM imidazole in the lysis buffer. Fractions containing protein were then pooled and loaded onto a HiPrep 26/10 Desalting column equilibrated with lysis buffer (AKTA purifier). Protein fractions were collected, and the purity of the protein was shown to be >95% by SDS-PAGE. The concentration of Dreiklang was determined by absorbance spectroscopy using ε280 = 21,890 M^-1^ cm^-1^. Proteins in the lysis buffer were lyophilized and resuspended in Tris buffer (20 mM Tris pH 6.5 150 mM NaCl) for TA or D_2_O Tris buffer (pD 6.5) for the FTIR or TRMPS measurements.

## **Figures**

Figure S1: Decay-associated difference spectra (DADS) obtained from global analysis of the OFF-state (cisHOH) transient absorption data using a parallel decay model. The spectra reveal a sub-picosecond rise in the excited-state absorption (ESA) band, followed by a two-step excited-state decay with time constants of 45 ps and 410 ps, ultimately forming a species that absorbs at 400 nm and persists until the end of the measurement window.

# Figure S2: **Kinetic traces at 500 nm from transient absorption measurements in H₂O (pH 6.5) and D₂O (pD 6.5), shown overlaid for comparison.**

# Figure S3: The EADS recovered for TRIR when the sub ps component from TA is imposed. No new spectral features are revealed.

# Figure S4: Decay-associated difference spectra (DADS) obtained from global analysis of the OFF-state (cis^HOH^) transient absorption data using a parallel decay model.

## **References**

[1] P. Roy, G. Bressan, J. Gretton, A. N. Cammidge, S. R. Meech, *Angew. Chemie - Int. Ed.* **2021**, *60*, 10568–10572.

[2] M. Towrie, D. C. Grills, J. Dyer, J. A. Weinstein, P. Matousek, R. Barton, P. D. Bailey, N. Subramaniam, W. M. Kwok, C. Ma, D. Phillips, A. W. Parker, M. W. George, *Life Sci. Leg. Georg. Porter* **2006**, *57*, 454–467.

[3] G. M. Greetham, D. Sole, I. P. Clark, A. W. Parker, M. R. Pollard, M. Towrie, *Rev. Sci. Instrum.* **2012**, *83*, DOI 10.1063/1.4758999.
